# Pharmacodynamic evaluation of suppression of in vitro resistance in *Acinetobacter baumannii* strains using polymyxin B-based combination therapy

**DOI:** 10.1038/s41598-021-90709-2

**Published:** 2021-05-31

**Authors:** Nayara Helisandra Fedrigo, Danielle Rosani Shinohara, Josmar Mazucheli, Sheila Alexandra Belini Nishiyama, Floristher Elaine Carrara-Marroni, Frederico Severino Martins, Peijuan Zhu, Mingming Yu, Sherwin Kenneth B. Sy, Maria Cristina Bronharo Tognim

**Affiliations:** 1 Laboratório de Microbiologia, Departamento de Ciências Básicas da Saúde, Universidade Estadual de Maringá, Avenida Colombo 5790, Maringá, Paraná CEP 87020-900 Brazil; 2 Department of Statistics, State University of Maringá, Maringá, Paraná Brazil; 3 Department of Pathology, Clinical and Toxicological Analysis, University Hospital, State University of Londrina, Londrina, Paraná Brazil; 4 esqLABS GmbH, Hambierich 34, 26683 Saterland, Germany; 5 Alexion Pharmaceuticals, Boston, MA USA; 6 School of Medicine and Pharmacy, Ocean University of China, Qingdao, Shandong China

**Keywords:** Microbiology, Therapeutics

## Abstract

The emergence of polymyxin resistance in Gram-negative bacteria infections has motivated the use of combination therapy. This study determined the mutant selection window (MSW) of polymyxin B alone and in combination with meropenem and fosfomycin against *A. baumannii* strains belonging to clonal lineages I and III. To evaluate the inhibition of in vitro drug resistance, we investigate the MSW-derived pharmacodynamic indices associated with resistance to polymyxin B administrated regimens as monotherapy and combination therapy, such as the percentage of each dosage interval that free plasma concentration was within the MSW (%T_MSW_) and the percentage of each dosage interval that free plasma concentration exceeded the mutant prevention concentration (%T_>MPC_). The MSW of polymyxin B varied between 1 and 16 µg/mL for polymyxin B-susceptible strains. The triple combination of polymyxin B with meropenem and fosfomycin inhibited the polymyxin B-resistant subpopulation in meropenem-resistant isolates and polymyxin B plus meropenem as a double combination sufficiently inhibited meropenem-intermediate, and susceptible strains. T_>MPC_ 90% was reached for polymyxin B in these combinations, while %T_MSW_ was 0 against all strains. T_MSW_ for meropenem and fosfomycin were also reduced. Effective antimicrobial combinations significantly reduced MSW. The MSW-derived pharmacodynamic indices can be used for the selection of effective combination regimen to combat the polymyxin B-resistant strain.

## Introduction

Polymyxins are an old class of antibiotics first discovered in the 1950s, but have regained interest over the past few years due to the emergence of multidrug-resistant Gram-negative bacteria (MDR-GNB) worldwide, particularly the carbapenem-resistant *Acinetobacter baumannii*, CR-Ab^1^. Although in recent years, the pharmaceutical industry with government initiatives has launched several new drugs in the market with activity against MDR-GNB; none of them, except cefiderocol and eravacycline, have antimicrobial activity against CR-Ab^2^.

The clinical use of polymyxin B is not thoroughly understood owing to the insufficient information on efficacy at the site of infection, pharmacokinetic limitation (potential nephrotoxicity) and the lack of clinically relevant susceptibility breakpoints^3,4^. The Clinical and Laboratory Standards Institute (CLSI) recently updated polymyxin B breakpoints only for intermediate (≤ 2 µg/mL) and resistant (≥ 4 µg/mL) interpretative categories, whereas European Committee on Antimicrobial Susceptibility Testing (EUCAST) maintained this interpretative category (susceptible ≤ 2 and resistant > 4 µg/mL)^5,6^. Polymyxin B demonstrates in vitro activity against CR-Ab^7,8^, however its use as monotherapy often results in clinical failure and emergence of drug resistance in the microorganism during therapy^9,10^, in part because therapeutic concentrations that kill the majority of susceptible cells may be insufficient to restrict the growth of resistant mutant subpopulation^11^.

The mutant selection window (MSW) hypothesis postulates this critical concentration range where resistant mutant subpopulation is selectively amplified. The upper limit of the window is the mutant prevention concentration (MPC) and the lower boundary is the minimum inhibitory concentration (MIC)^12^. According to this hypothesis, keeping drug concentrations above MPC throughout therapy will restrict the emergence of resistance and achieve its therapeutic effect. The high bacterial inocula used in the MPC measurements are also more representative of the bacterial burden that is present at the infection sites, such as pneumonia^13^. Given that polymyxin B has a very narrow therapeutic window, administration of this drug as monotherapy failed to attain MPC safely, especially in patients with renal impairment^14^.

Even though clinical data that explored the advantages of polymyxins combination therapy versus monotherapy presented conflicting results^15^, combination therapy is still relevant because the use of less-toxic and broad-spectrum antibiotics can restore polymyxin B activities^10,16–18^. Several in vitro studies demonstrated the synergistic effect of combinations with meropenem and fosfomycin^19–21^. Fosfomycin acts by blocking the MurA enzyme that is involved in the first steps of the peptidoglycan biosynthetic pathway^22^. Meropenem binds to penicillin-binding proteins in the periplasmic space, preventing peptidoglycan biosynthesis and viable cell wall production^23^. Polymyxins are cationic agents that bind to the anionic lipopolysaccharide and phospholipids molecules in the outer membrane, leading to a detergent-type effect that disrupts membrane integrity^24^. Concomitant use of antimicrobials acting on different cellular targets can theoretically result in the synergism between them and possibly restrict the selection of polymyxin B-resistant mutant bacteria.

There is an urgent need to investigate the effects of drug combination with the usual polymyxin B regimens on the resistant mutant subpopulation of both susceptible and non-susceptible strains. The current study determines the MPC of polymyxin B against *A. baumannii* and investigates the effect of combination therapy on pharmacodynamic indices associated with resistance suppression.

## Results

### Sequence type distribution of isolates and in vitro antimicrobial susceptibility

**Figure 1:**
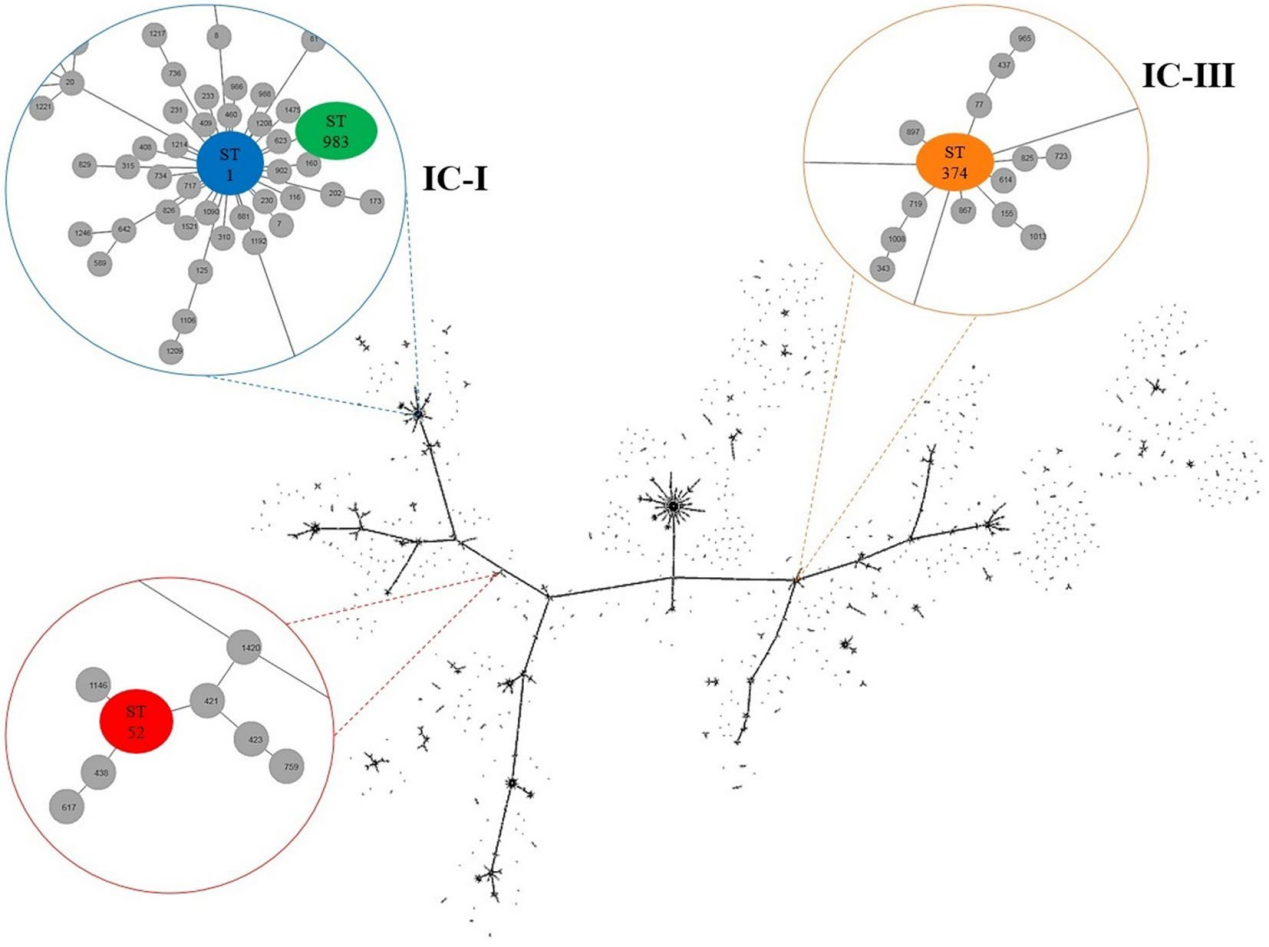
Diagram constructed by the goeBURST algorithm and using the Phyloviz software version 2.0 (http://www.phyloviz.net/) indicating the similarity among sequence type (ST) and international clone (IC) corresponding to *A. baumannii* clinical isolates studied. Each line indicates that the connected circles correspond to STs sharing 6/7 of the alleles. The STs observed in the present study are enlarged and highlighted in color.

Antimicrobial susceptibility profiles and molecular characteristics of all strains are described in Table 1. The population structure of the 1,557 sequence types available in the PubMLST *A. baumannii* database is shown in Fig. 1 (data accessed on December 8, 2020), based on the Pasteur scheme using the goeBURST algorithm implemented in Phyloviz Online^25^.

**Table 1:** Antimicrobial susceptibility profile and molecular characteristics of selected *A. baumannii* strains.

ID code	Strain name	Year	MLST	Non-susceptible antimicrobial profile	MIC/MPC in µg/mL			Carbapenemase genes
					PMB	MEM	FOF	
ATCC 19606	**–**	1948	ST52	AMP, AMC, CFZ, FOX, NIT, STX	1/4	2/16	128/2048	*–*
Ac56-HUEL	Ac-PMBs/Mi	1996	ST374	TZP, CAZ, FEP, CIP, LEV, CN, AMK	2/8	4/32	256/2048	*–*
Ac56-HUM	Ac-PMBs/Mr	2012	ST983	TZP, SAM, CAZ, FEP, CIP, LEV, GEN, AMK, IPM, MEM, SXT, TGC	2/16	16/64	128/2048	*bla*_VIM-like_
Ac576-HUEL	Ac-PMBr/Mr	2011	ST1	TZP, SAM, CAZ, FEP, CIP, LEV, GEN, AMK, IPM, MEM, SXT, TGC, PMB	32/64	64/ > 512	128/2048	*bla*_OXA-23_^a^

Susceptibility antimicrobial profile results were performed by automated systems. CLSI 2019 *A. baumannii* breakpoints (µg/mL) for PMB: susceptible (S) ≤ 2 and resistant (R) ≥ 4; and MEM: S ≤ 2, intermediate 4, R ≥ 8. EUCAST 2020 *A. baumannii* breakpoints (µg/mL) for colistin: S ≤ 2 and R > 2; and MEM: S ≤ 2 and R ≥ 8. FOF breakpoints (µg/mL) for Enterobacteriales according to the CLSI 2019 guidelines: S ≤ 64, intermediate 128, R ≥ 256.

*MLST* multilocus sequence typing, *ST* sequence type, *AMP* ampicillin, *AMC* amoxicillin-clavulanic acid, *CFZ* cefazolin, *FOX* cefoxitin, *NIT* nitrofurantoin, *SXT* trimetoprim-sulfamethoxazole, *TZP* piperacillin-tazobactam, *SAM* ampicillin-sulbactam, *CAZ* ceftazidime, *FEP* cefepime, *IPM* imipenem, *MEM* meropenem, *CIP* ciprofloxacin, *LEV* levofloxacin, *AMK* amikacin, *GEN* gentamicin, *PMB* polymyxin B, *TGC* tigecycline, *FOF* fosfomycin.

^a^*bla*_OXA-23-like_ carbapenemase gene is associated with the strong promoter IS*Aba1* located upstream of the gene.

**Figure 2:**
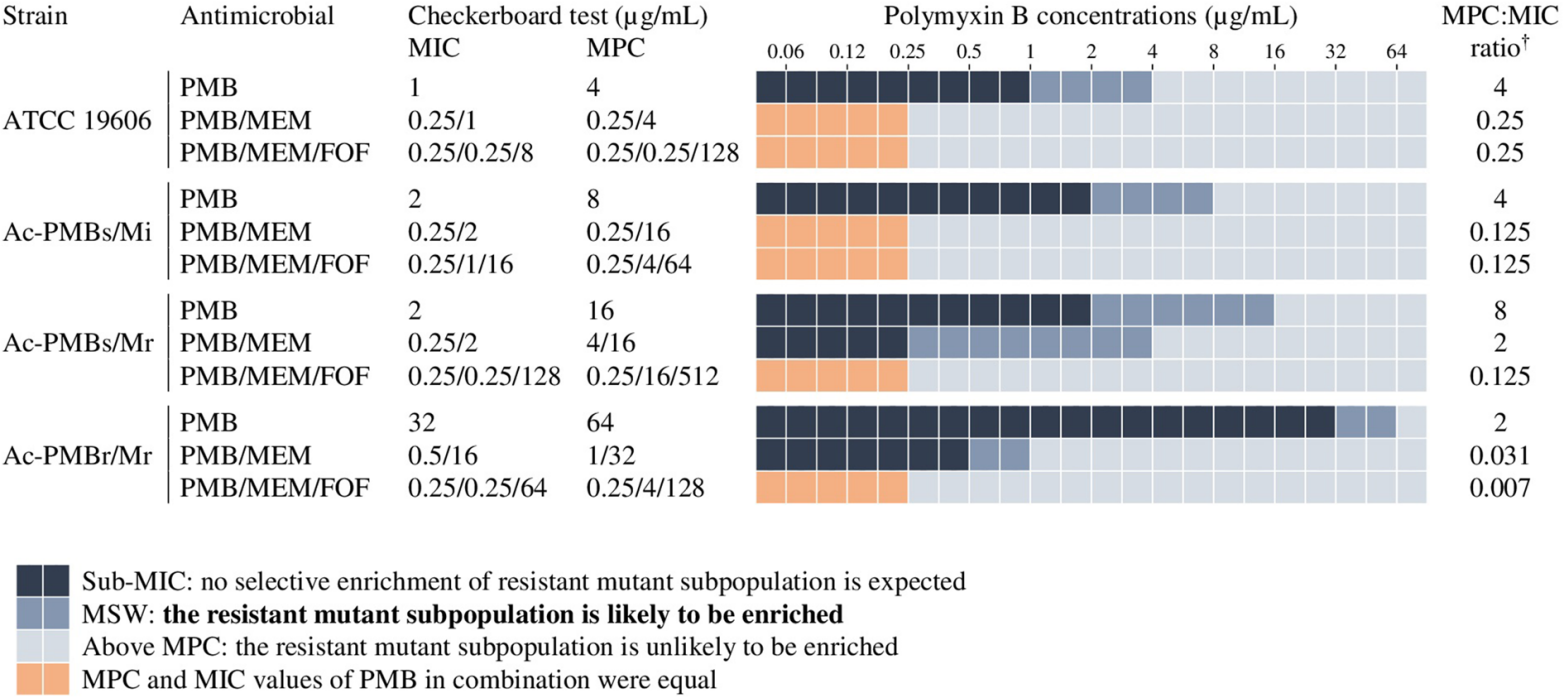
MIC, MPC, and MIC/MPC ratio of polymyxin B alone and in combination with meropenem and fosfomycin against *A. baumannii* strains. *MIC* minimum inhibitory concentration, *MPC* mutant prevention concentration, *MSW* mutant selection window, *PMB* polymyxin B, *MEM* meropenem, *FOF* fosfomycin, *PMB/MEM* PMB combined with MEM, *PMB/MEM/FOF* the combination of all three antimicrobials. ^†^MPC:MIC ratio is defined as the ratio of the MPC obtained to the original MIC and smaller values indicate a better ability to prevent the emergence of mutants.

The MIC, MPC, and MPC:MIC ratio of polymyxin B alone and in combination with meropenem and fosfomycin for each strain are shown in Fig. 2. For Ac-PMBs/Mi isolate, the MPC value was four times higher than MIC (MSW from 2 to 8 µg/mL). The ATCC 19606 strain had similar MSW compared with this clinical isolate. The Ac-PMBs/Mr showed a wide MSW covering concentrations between 2 and 16 µg/mL. On the other hand, a narrow MSW was observed for Ac-PMBr/Mr isolate, whose concentration ranged between 32 and 64 µg/mL.

We evaluated the hypothesis that effective antimicrobial combinations will reduce or even close the MSW of polymyxin B, meropenem and fosfomycin. The MICs and MPCs were lower than those obtained from polymyxin B alone for all strains (Fig. 2). After the addition of meropenem, both MIC and MPC values decreased up to thirty-two times for polymyxin B (0.25 µg/mL) against ATCC 19606 and Ac-PMBs/Mi strains, closing the MSW and conferring susceptibility to polymyxin B based on the breakpoint value of ≤ 2 µg/mL^6,26^. The MPC:MIC ratio indicated that polymyxin B in combination with meropenem restricted the emergence of polymyxin B resistance against these strains. For Ac-PMBs/Mr isolate, a MSW varying between 0.25 and 4 µg/mL was maintained even when polymyxin B was combined with meropenem. The Ac-PMBr/Mr showed a greater reduction of MIC and MPC values (sixty-four times) with the two-agent combination than the polymyxin B-susceptible strains, resulting in attainable plasma levels for polymyxin B (MIC ≤ 2 µg/mL), despite a narrow MSW ranging between 0.5 and 1 µg/mL was present.

The addition of fosfomycin in combination with polymyxin B plus meropenem further decreased the MIC and MPC values of polymyxin B to 0.25 µg/mL against Ac-PMBs/Mr and Ac-PMBr/Mr isolates, closing the MSW and significantly restricting the selective amplification of polymyxin B-resistant subpopulation (Fig. 2). There was a greater effect of the two drugs on MPC:MIC ratio of polymyxin B than that tested individually. Furthermore, the three-drug combination recovered the carbapenem susceptibility with MICs ≤ 2 µg/mL for these strains^6,26^. Only one out of four strains did not show a reduction in MPC of meropenem in the triple combination (Ac-PMBs/Mr).

### Pharmacokinetic/pharmacodynamic (PK/PD) analysis and suppression of resistance

Table 2 shows the PK/PD analysis for five dosing regimens of polymyxin B in monotherapy and combined with meropenem and fosfomycin against four strains. None of the polymyxin B monotherapy regimens achieved plasma concentrations sufficient to attain at least a MIC of 32 µg/mL for Ac-PMBr/Mr, whereas, for the polymyxin B-susceptible strains, a T_MSW_ up to 68% of the dosing interval was observed.

**Table 2:** MSW-derived PD indices of polymyxin B alone and in combination against *A. baumannii* strains.

Strain	Polymyxin B dosage regimen^a^	Monotherapy		Double-combination of polymyxin B regimen/2 g q8h meropenem^b^		Triple-combination of polymyxin B regimen/2 g q8h meropenem/8 g q8h fosfomycin^b^	
		%T_MSW_^c^	%T_>MPC_^c^	%T_MSW_	%T_>MPC_	%T_MSW_	%T_>MPC_
ATCC 19606	1 mg/kg q12h	12.5 ± 12.7	0.23 ± 1.26	0	48.7 ± 19.3	0	48.7 ± 19.3
	1.5 mg/kg q12h	68.1 ± 31.1	14.5 ± 24.8	0	96.0 ± 11.5	0	96.0 ± 11.5
	LD plus 0.5 mg/kg q12h	22.0 ± 12.7	2.94 ± 4.82	0	56.9 ± 19.7	0	56.9 ± 19.7
	LD plus 1 mg/kg q12h	57.5 ± 29.5	6.75 ± 14.5	0	97.6 ± 6.7	0	97.6 ± 6.7
	LD plus 100 mg q12h	27.6 ± 13.6	4.25 ± 6.6	0	64.4 ± 19.4	0	64.4 ± 19.4
Ac-PMBs/Mi	1 mg/kg q12h	10.8 ± 16.2	NC	0	94.1 ± 15.7	0	94.1 ± 15.7
	1.5 mg/kg q12h	24.4 ± 25.8	0.1 ± 1.1	0	98.2 ± 8.08	0	98.2 ± 8.08
	LD plus 0.5 mg/kg q12h	34.1 ± 31.2	1.2 ± 4.8	0	99.3 ± 4.8	0	99.3 ± 4.8
	LD plus 1 mg/kg q12h	40.3 ± 33.9	1.3 ± 4.2	0	99.6 ± 3.5	0	99.6 ± 3.5
	LD plus 100 mg q12h	44.5 ± 34.3	1.5 ± 4.6	0	99.8 ± 2.6	0	99.8 ± 2.6
Ac-PMBs/Mr	1 mg/kg q12h	10.8 ± 16.3	NC	93.3 ± 15.8	0.8 ± 3.3	0	94.1 ± 15.7
	1.5 mg/kg q12h	24.5 ± 26.1	NC	93.6 ± 12.1	4.7 ± 9.8	0	98.2 ± 8.1
	LD plus 0.5 mg/kg q12h	35.4 ± 32.9	NC	89.2 ± 16.3	10.1 ± 16.0	0	99.3 ± 4.8
	LD plus 1 mg/kg q12h	41.7 ± 35.5	NC	87.2 ± 19.6	12.4 ± 19.4	0	99.6 ± 3.5
	LD plus 100 mg q12h	46.0 ± 35.8	NC	85.4 ± 20.8	14.3 ± 20.7	0	99.8 ± 2.6
Ac-PMBr/Mr	1 mg/kg q12h	NC	NC	35.2 ± 21.4	37.5 ± 31.6	0	94.1 ± 15.7
	1.5 mg/kg q12h	NC	NC	29.2 ± 23.3	58.5 ± 33.8	0	98.2 ± 8.1
	LD plus 0.5 mg/kg q12h	NC	NC	22.1 ± 25.0	71.6 ± 32.3	0	99.3 ± 4.8
	LD plus 1 mg/kg q12h	NC	NC	18.2 ± 26.0	77.9 ± 31.1	0	99.6 ± 3.5
	LD plus 100 mg q12h	NC	NC	15.5 ± 24.8	81.7 ± 29.0	0	99.8 ± 2.9

*MSW* mutant selection window, *MPC* mutant prevention concentration, *LD* loading dose of 3.0 mg/kg, *NC* not computable because polymyxin B MIC is higher than maximum. Values reported as mean ± SD.

^a^All regimens were 1-h infusions.

^b^Meropenem and fosfomycin regimens were administered as 3-h prolonged infusions.

^c^T_MSW_ or T_>MPC_ was calculated as the percentage of the first 24 h.

**Figure 3:**
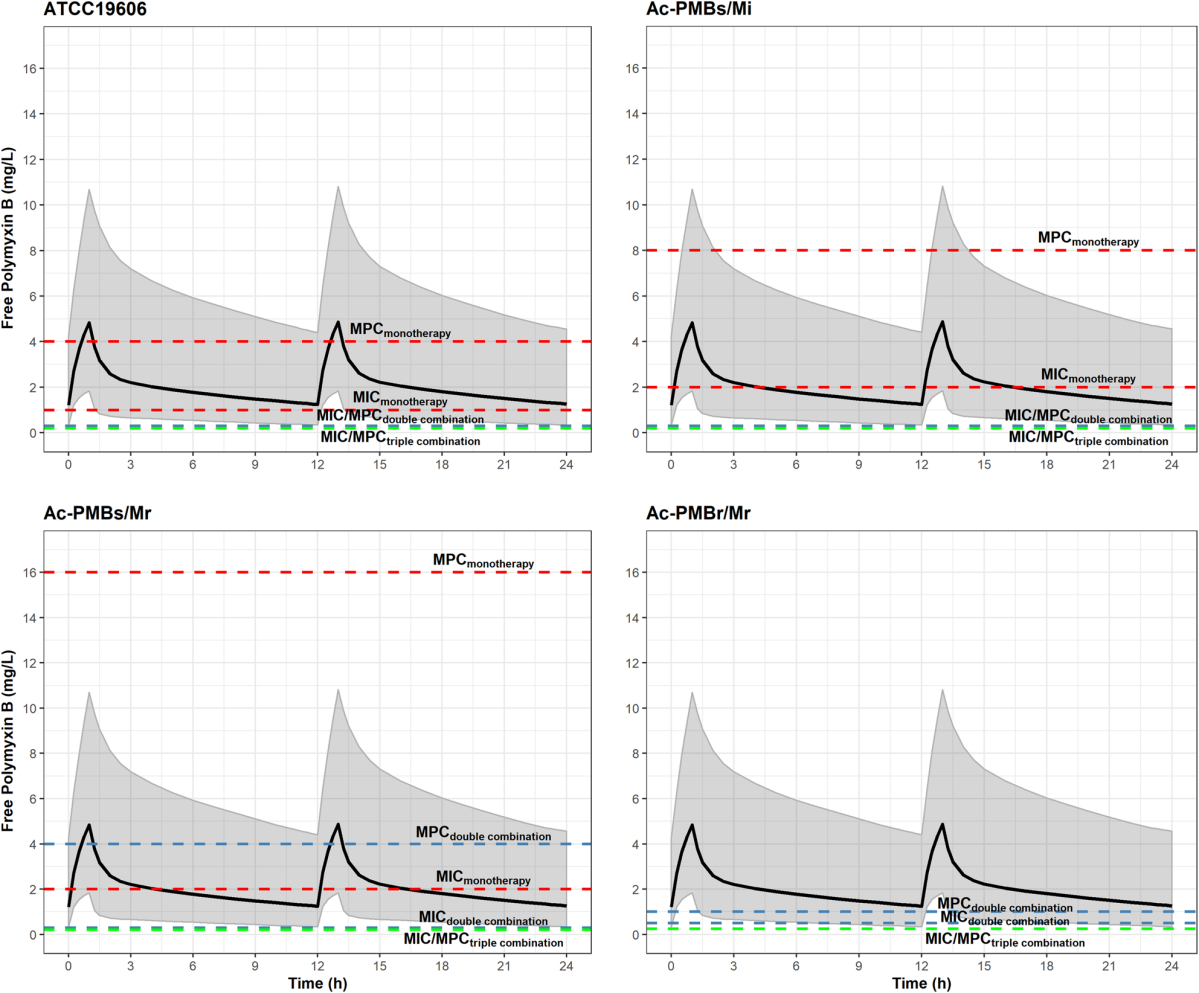
Simulated median and 95% prediction interval of free drug concentrations, as represented by solid black lines and grey shaded areas, respectively, in virtual patients against *A. baumannii* strains. The dosing regimen of polymyxin B at 1.5 mg/kg q12h with 1-h infusion was simulated in 10,000 virtual patients. MIC and MPC values in monotherapy (red), two- (blue), and three-drug combination (green) are shown by dashed lines.

All polymyxin B dose regimens as double combination reduced the T_MSW_ to 0% against ATCC 19606 and Ac-PMBs/Mi strains, while plasma concentrations were above the MPC of 0.25 µg/mL for a duration of at least 96% of the dosing interval at 1.5 mg/kg q12h (Table 2 and Fig. 3). Greater than 87% T_MSW_ was maintained for Ac-PMBs/Mr isolate even with the higher dose of 3 mg/kg followed by 1 mg/kg q12h. Concentrations of polymyxin B fell within the MSW in all dose regimens against Ac-PMBr/Mr isolate, although a reasonable T_>MPC_ up to 78% of the dosing interval was achieved at 3 mg/kg followed by 1 mg/kg q12h.

The triple combination provided a T_MSW_ of 0% for all strains, including those resistant to carbapenem and polymyxin B (Ac-PMBs/Mr and Ac-PMBr/Mr), in the five polymyxin B dosage regimens tested. Similarly, polymyxin B concentrations remained above 98% T_>MPC_ against both Ac-PMBs/Mr and PMBr/Mr isolates at 1.5 mg/kg q12h, which covered 99.13% of the virtual population free concentrations above 0.25 µg/mL (Fig. 3). There was no difference in T_>MPC_ between using 1.5 mg/kg q12h and the high dose of 3.0 mg/kg followed by 1 mg/kg at q12h (Table 2).

The polymyxin B regimen consisting of a loading dose (3 mg/kg) followed by a fixed dose of 100 mg q12h produced T_MSW_ and T_>MPC_ similar to those achieved with the regimen consisting of a loading dose plus a weight-based dose of 1 mg/kg at q12h for the four isolates tested (Table 2).

Table 3 shows the T_MSW_ and T_>MPC_ achieved by the monotherapy and combination therapy for meropenem and fosfomycin in various dosing regimens as 3-h infusions. None of the fosfomycin monotherapy regimens provided plasma concentrations sufficient to achieve the MIC against all strains (≥ 128 µg/mL) (Fig. 2). On the other hand, when used in the triple combination, fosfomycin concentrations remained above the MPC of 64 µg/mL for more than 70% of the dosing interval at the high dose of 8 g q8h for all strains, except Ac-PMBs/Mr for which a T_MSW_ up to 57% of the dosing interval was observed.

**Table 3:** MSW-derived PD indices of meropenem and fosfomycin as monotherapy and in combination against *A. baumannii* strains.

Strain	Antimicrobial dosage regimen^a^	Monotherapy		Combination therapy			
		%T_MSW_^†^	%T_>MPC_^†^	%T_MSW_	%T_>MPC_	%T_MSW_	%T_>MPC_
	*Meropenem*			Double-combination with LD plus 1 mg/kg q12h polymyxin B^b^		Triple-combination with 8 g q8h fosfomycin/LD plus 1 mg/kg q12h polymyxin B	
ATCC 19606	1 g q8h	55.6 ± 16	40.5 ± 16.7	2.3 ± 3.6	97.3 ± 3.6	0	100 ± 0
	2 g q8h	14.6 ± 12.9	84.9 ± 12.9	0.7 ± 0.6	99.0 ± 0.6	0	100 ± 0
Ac-PMBs/Mi	1 g q8h	93.4 ± 8.9	0.10 ± 0.99	68.7 ± 16.6	29.7 ± 17.4	5.9 ± 8.6	93.6 ± 8.9
	2 g q8h	68.7 ± 16.6	29.7 ± 17.4	24.8 ± 16.8	74.6 ± 17.0	1.3 ± 3.0	98.5 ± 3.1
Ac-PMBs/Mr	1 g q8h	29.7 ± 17.4	0	68.7 ± 16.6	29.7 ± 17.4	70.2 ± 17.4	29.7 ± 17.4
	2 g q8h	74.5 ± 17.0	0.10 ± 0.99	24.8 ± 16.8	74.6 ± 17.0	25.3 ± 17.0	74.6 ± 17.0
Ac-PMBr/Mr	1 g q8h	NC	NC	29.6 ± 17.2	0.10 ± 0.99	6.2 ± 9.5	93.6 ± 8.9
	2 g q8h	NC	NC	44.9 ± 6.1	29.7 ± 17.4	1.5 ± 3.1	98.5 ± 3.1

	*Fosfomycin*					Triple-combination with 2 g q8h meropenem/LD plus 1 mg/kg q12h polymyxin B	
ATCC 19606	4 g q8h	NC	NC	ND	ND	54.8 ± 44.8	43.2 ± 45.9
	6 g q8h	NC	NC	ND	ND	38.4 ± 42.7	60.4 ± 43.9
	8 g q8h	NC	NC	ND	ND	27.8 ± 37.8	71.3 ± 39.0
Ac-PMBs/Mi	4 g q8h	NC	NC	ND	ND	24.2 ± 32.8	71.3 ± 39.0
	6 g q8h	NC	NC	ND	ND	14.4 ± 24.4	82.9 ± 30.1
	8 g q8h	NC	NC	ND	ND	9.5 ± 19.0	88.6 ± 24.0
Ac-PMBs/Mr	4 g q8h	NC	NC	ND	ND	42.6 ± 45.5	0.62 ± 6.3
	6 g q8h	NC	NC	ND	ND	54.6 ± 43.8	5.8 ± 21.2
	8 g q8h	NC	NC	ND	ND	57.1 ± 42.1	14.2 ± 32.6
Ac-PMBr/Mr	4 g q8h	NC	NC	ND	ND	28.0 ± 33.9	43.2 ± 45.9
	6 g q8h	NC	NC	ND	ND	22.5 ± 25.5	60.4 ± 43.9
	8 g q8h	NC	NC	ND	ND	17.3 ± 24.0	71.3 ± 39.0

*MSW* mutant selection window, *MPC* mutant prevention concentration, *LD* loading dose of 3.0 mg/kg, *NC* not computable because polymyxin B MIC is higher than maximum. *ND* not tested. Values reported as mean ± SD.

^a^Meropenem and fosfomycin regimens were administered as 3-h prolonged infusions.

^b^Polymyxin B regimen were 1-h infusions.

^c^T_MSW_ or T_>MPC_ was calculated as the percentage of the first 24 h.

The meropenem regimens evaluated as monotherapy provided reasonable T_>MPC_ only for the ATCC 19606 strain that exhibited MPC of 0.25 µg/mL; the regimen of 2 g q8h resulted in a T_>MPC_ of 85% (Table 3 and Fig. 2). Meropenem as double combination achieved 75% T_>MPC_ against Ac-PMBs/Mi and Ac-PMBs/Mr isolates at the same dosing regimen, whereas a T_MSW_ of 25% was observed within the concentration range between MIC of 2 µg/mL and MPC of 16 µg/mL. The triple combination provided the T_>MPC_ of 98% against Ac-PMBr/Mr at 4 µg/mL MPC for the meropenem regimen of 2 g q8h, which was also observed for the Ac-PMBs/Mi isolate. Only ATCC 19606 strain presented T_MSW_ of 0%, while meropenem concentrations remained above the MPC over the entire dosing interval in both simulated regimens. Ac-PMBs/Mr isolate did not show an increase in T_>MPC_ of meropenem in the triple combination.

## Discussion

Polymyxin B is one of the few remaining options for the treatment of CR-Ab, but its use in monotherapy has led to the emergence of resistant strains^9–11^. To the best of our knowledge, this study is the first to report the MPC and MSW results for polymyxin B against *A. baumannii* strains and to evaluate the duration of time that polymyxin B levels remain above the MPC during monotherapy or combination therapy regimens, as well as whether combination therapy reduces the duration of MSW. Our results showed that the combination of polymyxin B with meropenem and fosfomycin inhibited the in vitro growth of polymyxin B-resistant subpopulation and significantly lowered the MPC of polymyxin B (0.25 µg/mL).

Three clinical isolates of *A. baumannii* with different sequence types and susceptibility profiles to polymyxin B and meropenem were selected in order to investigate the emergence of polymyxin resistance in MDR clonal lineages, or international clones, that are spreading around the world^27,28^. Several in vitro studies using colistin have shown that its MSW varied between 0.5 and ≥ 128 µg/mL for colistin-susceptible MDR *A. baumannii* isolates^29–31^. Our findings demonstrated that the MSW of polymyxin B against these isolates is not similar to that of colistin. Polymyxin is the treatment of choice for these isolates but its use as monotherapy should be avoided due to the resistant mutants that may be selectively amplified.

Interestingly, metallo-β-lactamase-producing carbapenem-resistant isolate (Ac-PMBs/Mr) had the widest MSW of polymyxin B (2 and 16 µg/mL). This phenomenon can be partially attributed to the presence of mobile genetic elements such as transposons contained in certain regions of the genome which favor modifications of the lipopolysaccharide and can lead to polymyxin resistance^32,33^. VIM-type metallo-β-lactamase is often encoded on mobile gene cassettes inserted into class 1 integron, which can be found as part of transposons^34^. In the case of oxacillinase-producing isolate (Ac-PMBr/Mr), although the bacterial population was composed predominantly of polymyxin B-resistant cells (MIC 32 µg/mL), a subpopulation with a higher degree of resistance was detected at concentrations up to 64 µg/mL, as indicated by MPC testing. This may be associated with virulence factors such as its remarkable ability to adhere and survive in hospital surfaces already identified in a previous study^35^. Both carbapenemase-producing CR-Ab had STs that belonged to clonal complex 1 and international clone I, which presents a broad international distribution among several countries, including Brazil (Fig. 1)^27,36^. Conversely, Ac-PMBs/Mi isolate is an ancient lineage assigned to ST374, belonging to clonal complex 3 and international clone III, that was reported in few countries to date^28^ (PubMLST.org databases) and shown similar behavior to the ATCC 19606 strain.

The PK/PD evaluation shows that a high daily dose of polymyxin B results in an average steady-state plasma drug concentration of ≥ 2 µg/mL, which is expected to be efficacious against an organism with a MIC ≤ 2 µg/mL^37^; however, this exposure of polymyxin B alone may not be sufficient to achieve MPC, as shown in our study*.* In order to optimize dosages with respect to minimizing resistance development, we estimated two PK/PD indices, T_>MPC_ and T_MSW,_ based on the MPC concept and the emergence of resistance. The results of the simulation showed that higher polymyxin B exposures in monotherapy increased the percentage of time during which polymyxin B stays within the MSW (T_MSW_) and the opportunity for resistant subpopulation to be selectively enriched in polymyxin B-susceptible strains based on MIC. In a recent in vitro hollow-fiber infection model of *A. baumannii*, Tsuji et al*.* also demonstrated that increasing the dose of polymyxin B in monotherapy amplified polymyxin B-resistant subpopulation^11^. Thus, increasing polymyxin B dose is not beneficial in suppressing resistance development and maximizing therapeutic effect, especially because polymyxins present a very narrow therapeutic window^14^.

Combination therapy is generally an effective strategy to prevent antimicrobial resistance, although clinical data regarding the advantage of combination therapy over polymyxin monotherapy are limited^38^. We investigated whether combining polymyxin B with a second or third bactericidal antibiotic could reduce the duration that polymyxin B levels stay in MSW while maximizing the duration that polymyxin B levels remain above the MPC. Among the polymyxin B-susceptible strains, with the exception of Ac-PMBs/Mr, the combination of polymyxin B plus meropenem resulted in the reduction of MIC and MPC values of polymyxin B to 0.25 µg/mL, closing the MSWs, while MIC of meropenem decreased to values close to those of the susceptible phenotypes (≤ 2 µg/mL). For carbapenemase-producing isolates, there were two new MSWs of the combination polymyxin B plus meropenem; the resistant mutant subpopulation is likely to become enriched when the concentrations of polymyxin B and meropenem fall within their MSW in the combination therapy. The complete suppression of resistance occurs when the combination closes all the original MSW of each antimicrobial. However, it is difficult to find a combination that can achieve this. When fosfomycin was added to the combination, the MIC and MPC of polymyxin B decreased to 0.25 µg/mL, closing the MSW and allowing that a lower dose of polymyxin B to be used in the therapy. The triple combination also improved the activity of meropenem and fosfomycin against carbapenemase-producing bacteria, with the exception of Ac-PMBs/Mr for which the MPC of the carbapenem was 16 µg/mL either in the double or triple combination.

*A. baumannii* is naturally resistant to a number of antimicrobials commonly used in the clinical practice against GNB, including fosfomycin^26^. However, fosfomycin presents a characteristic that makes it an attractive option as a partner drug for combinations, such as negligible plasma protein binding, very low toxicity and excellent distribution throughout the fluid and tissues in the body^22^. In our study, fosfomycin concentrations needed to close the MSW of polymyxin B ranged from 64 to 512 µg/mL. Previous in vivo studies showed that, in general, the serum concentration of fosfomycin was high, reaching a peak of up to 606 mg/L after intravenous administration^22^, which would probably prevent the emergence of polymyxin B-resistant mutants during treatment. Furthermore, others factors need to be taken into account in the antimicrobial activity and the suppression of resistance including specific characteristics of patient and pathogen.

Our simulation results showed that a dosage of 1.5 mg/kg q12h can maintain polymyxin B concentrations above the MPC during > 90% of the dosing interval (T_>MPC_ > 90%) given a MPC of 0.25 µg/mL for polymyxin B in combination. Given the %T_MSW_ is 0, the emergence of polymyxin B-resistant subpopulation may be restricted using triple combination therapy against CR-Ab in all dosage regimens tested. For carbapenem and polymyxin B non-resistant strains, the double combination would be sufficient. Although the original MSWs of meropenem and fosfomycin were not closed, the duration of time during which drug concentration stayed within their MSW was reduced in combination therapy. The assumption for the low dose of polymyxin B, i.e. without a loading dose, is to restrict the emergence of resistance and at the same time minimize toxicity, because loading dose is often used in serious infections when the MICs are not known prior to the initiation of the first dose. MSW-based PK/PD indices for polymyxin that relate to the suppression of resistance are largely unknown. Additional studies will be necessary to determine the variables that best predict resistance restriction in *A. baumannii*.

Most of the clinical studies for CR-Ab were associated with pneumonia infection^1^. Zaccard et al*.* showed that lung infections due to Gram-negative bacteria can have a bacterial burden as high as 10^8^ CFU^39^. Our findings demonstrated that polymyxin B in triple combination with meropenem and fosfomycin would be able to achieve the necessary exposure required for microbiological eradication of CR-Ab from the infected lung. Lenhard et al*.* in an in vitro hollow fiber study using the triple combination of sulbactam, meropenem and polymyxin B found a significant bactericidal activity for a high-density burden in the MDR *A. baumannii* strain, but the bacterial inoculum used in the checkerboard study was ~ 10^5^ CFU and did not take into account the probability of mutant subpopulation being present in the populations^10^. One limitation of the present study is that the bactericidal activity of polymyxin B was evaluated using culture a medium at pH 7.2, which is close to human blood. Some studies suggest that the body fluid pH, such as in the lung, may affect the therapeutic response to antibacterial agents and promote resistance^40,41^. The other limitation of the study is that the determinations of PD parameters were based on free plasma concentrations. The extension to efficacy in the epithelial lining fluid (ELF) is limited due to the lack of information on drug concentrations in the ELF of humans for polymyxin and fosfomycin, since these two antibiotics are very old. Meropenem penetration into the ELF of patients with ventilator-associated pneumonia is approximately 25% of the plasma drug concentration^42^. Fosfomycin exposure in ELF of weaning piglets was only 13% to that in the plasma^43^. Given the lower free drug concentrations in the ELF, the %T_MSW_ will likely be higher whereas T_>MPC_ will be lower.

In conclusion, this study reported the MPC and MSW results for polymyxin B and evaluated PK/PD indices that were related to the suppression of resistance in *A. baumannii*. The triple combination of polymyxin B, meropenem, and fosfomycin can close the MSW of polymyxin B in vitro against CR-Ab and maximize the duration of time that polymyxin B levels remain above the MPC in order to prevent the emergence of resistant mutants, enabling the use of this triple combination in clinical practice against this fearsome pathogen.

## Methods

### Bacterial samples

The surveillance and monitoring program of antimicrobial resistance in *Acinetobacter* spp. has been established in three tertiary hospitals in the southern Brazil since 1994 by the Medical Microbiology Laboratory at State University of Maringá. Identification and antimicrobial susceptibility profiles of bacterial isolates were performed by routine automated microbiology systems, such as Phoenix^®^ (BD Diagnostic Systems, Sparks, MD) and MicroScan® (DadeBehring, West Sacramento, CA) according to the equipment's instructions. These isolates were preserved at − 20 °C in Trypticase Soy Broth (Difco Laboratories, Detroit, MI) with 30% glycerol until they were tested for their susceptibility to antibiotics.

Molecular typing was performed using ERIC-PCR assays followed by an analysis of gels in Bionumerics^®^ v. 6.5 (Applied Maths, Sint-Martens-Latem, Belgium)^44^. The MIC values of polymyxin B and meropenem against *A. baumannii* were additionally tested by the broth microdilution method in CAMHB (Difco Laboratories, Sparks, MD) as described in CLSI document M07-A10^45^. Considering the similarity of the clusters (based on Dice correlation coefficient > 0.8) and dissimilar susceptibility patterns to polymyxin B and meropenem, eight clinical isolates (data not shown) were characterized using Pasteur MLST scheme available in the *A. baumannii* MLST website (https://pubmlst.org/organisms/acinetobacter-baumannii/)^46^.

Among these isolates, three *A. baumannii* belonging to different STs and antimicrobial susceptibility profiles were selected for this study. The MIC interpretive criteria for both polymyxin B and meropenem according to EUCAST^6^ and CLSI^26^ guidelines were used to name the isolates as: Ac-PMBs/Mi (*A. baumannii* polymyxin B susceptible/meropenem intermediate) with no carbapenemase enzymes; Ac-PMBs/Mr (*A. baumannii* polymyxin B susceptible/meropenem resistant) carrying *bla*_VIM-like_ metallo-β-lactamase gene that was detected by the multiplex PCR assay^47^; and Ac-PMBr/Mr (*A. baumannii* polymyxin B/meropenem resistant) which harbored the association of *bla*_OXA23-like_ carbapenemase gene with IS*Aba1* upstream^48^, but a *mcr-1* gene was not found^49^. Additionally, *A. baumannii* ATCC 19606 was included in the set of tests as a reference strain for antimicrobial quality control.

### MIC and MPC determination

#### Antimicrobial agents

Fosfomycin (Sigma-Aldrich, St. Louis, MO, USA) was purchased from LabCompany (Londrina, Paraná, Brazil). Polymyxin B (Eurofarma, Itapevi, São Paulo, Brazil) and meropenem (BioChimico, Itatiaia, Rio de Janeiro, Brazil) were kindly provided by the State University of Maringa Hospital. Fosfomycin and meropenem were dissolved in water to a final concentration of 10 mg/ml and stored at − 20 °C (stock solution) and polymyxin B solution was prepared at the same concentration on the day of experimentation.

#### Bacterial inoculum size

Two inoculum sizes of each strain were used in the experiments. Inoculum were prepared in tubes containing 3 mL of sterile Mueller–Hinton broth (MHB; Difco Laboratories, Detroit, MI) from an overnight culture on Mueller–Hinton agar (MHA; Difco Laboratories, Detroit, MI) plates at 35 ± 2 °C in ambient air.

For MIC measurements, four to five individual colonies appearing on the plate were added in MHB and the suspension adjusted to an equivalent of 0.5 McFarland standard, at a density of approximately 1.5 × 10^8^ CFU/mL, using a nephelometer (PhoenixSpec nephelometer; BD, Sparks, MD, USA)^45^. This suspension was further diluted in MHB resulting in a final inoculum of 5 × 10^5^ CFU/mL.

For MPC measurements, a final high-density inoculum of ≥ 10^9^ CFU/mL was used to ensure the emergence of the resistant mutant subpopulation^12^. In this case, the suspension was obtained from a carpet of colonies on the plate and the OD 660 nm was confirmed by absorbance reading with a spectrophotometer (Milton Roy Spectronic 21D spectrophotometer)^50^. Colony counts of the inoculum suspension were previously verified by counting CFU.

#### Determination of MICs and MPCs of single agents

The MIC and MPC of all three antimicrobials against each strain were determined in duplicate based on the method reported by Dong et al*.*, with modifications^12^. In brief, the bacterial inoculum suspensions corresponding to MIC and MPC tests were added to the 96-well U-bottom plate and inoculated simultaneously onto the surface of the drug-containing agar and drug-free control agar with a Steer's replicator. MHA plates containing fosfomycin was supplemented with 25 µg/mL of glucose-6-phosphate^45^. The concentrations ranges of polymyxin B, meropenem and fosfomycin were 0.25 to 256 µg/mL, 0.25 to 512 µg/mL and 2 to 2048 µg/mL, respectively.

#### MIC and MPC of antimicrobials in combinations

The antimicrobial activities of polymyxin B in combination with meropenem and fosfomycin against four *A. baumannii* strains were tested in duplicate by the checkerboard method^51^. For this test, the combinations analyzed included polymyxin B plus meropenem as double combination, and polymyxin B plus meropenem and fosfomycin as triple combination. The selected concentrations were 1/8 × , 1/4 × , 1/2 × , 1 × , 2 × , 4 × and 8 × MIC of both meropenem and fosfomycin, while polymyxin B concentrations ranged from 0.25 to 2 µg/mL representing clinically achievable free or unbound plasma levels in patients with severe infections^20,37^. Combinations containing fosfomycin were supplemented with 25 µg/mL glucose-6-phosphate^45^.

#### Interpretation of MIC, MPC and MSW

The MIC was defined as the lowest concentration of drug inhibiting the visible colony growth after 20 to 24 h of incubation^45^. The MIC results were interpreted according to the breakpoints established by the EUCAST and CLSI guidelines for *A. baumannii* strains^6,26^.

The MPC was defined as the lowest concentration of antimicrobial that prevented colony formation from a culture containing ≥ 10^9^ CFU bacteria. The plates were examined after the incubation period of 24 and 48 h^12^.

The antimicrobial concentrations range exceeding the MIC up to the MPC was defined as the MSW, at which selective amplification of antibiotic-resistant mutants occurs^52^.

The MPC:MIC ratio of polymyxin B alone and combined was also calculated and expressed as the ability of each combination to select resistant mutants. Lower values of the ratio indicate a better ability to restrict the emergence of resistant mutants^53^.

### Population PK in critically ill patients

Polymyxin B, meropenem and fosfomycin steady-state concentration–time profiles were simulated from population PK models. The individual PK parameters were assumed to be log-normally distributed.

The model for polymyxin B in critically ill patients was a two-compartment model^37^ parameterized with clearance of 0.0276 L/h/kg (CL; 32.4% CV), intercompartmental clearance of 0.146 L/h/kg (Q; 50.4% CV), central volume of distribution of 0.0939 L/kg (V_C_; 73.3% CV), and peripheral volume of 0.330 L/kg (V_P_; 70.1% CV). The final individual parameters were obtained by multiplying the body-weight scaled parameters with the patient’s body weight. The free drug concentration was computed by multiplying the simulated plasma drug concentration by 42%, wherein the plasma protein binding of polymyxin B was 58%. Four simulated weight-based polymyxin B regimens were 1 or 1.5 mg/kg every 12 h (q12h) and 3 mg/kg first dose followed by 0.5 and 1 mg/kg q12h. These dosing regimens were based on the most common regimens used in countries in which they were registered. Additionally, the 3 mg/kg first dose followed by a fixed-dose regimen of 100 mg q12h was also evaluated as previously proposed dosing regimen^54^. All regimens were administered as 1-h infusions.

The intravenous infusion model for meropenem was a one-compartment model previously shown to be more suitable for the prediction of free meropenem free concentrations in critically ill patients^55^. The model was parameterized with CL and V_C_. CL was dependent on serum creatinine (SCr) with the following relationship: ${\text{CL(L/h) = 11}}{.1} \times \left[ {\frac{{{\text{SCr}}}}{{{0}{\text{.7}}}}} \right]^{ - 1}$ with a CV of 52.1%. V_C_ was 33.6 L. The plasma protein binding of meropenem was 2%. The dosing regimens of meropenem evaluated were 1 g and 2 g q8h as prolonged infusion of 3 h.

The intravenous infusion model for fosfomycin was a two-compartment model; creatinine clearance (CLCr) was a covariate of clearance and body weight was a covariate of central volume, as previously described^56^ : ${\text{CL(L/h) = 5}}{.25} \times \left[ {\frac{{{\text{CLCr}}}}{{{90}}}} \right]$ with a CV of 91.9%; $V_{c} \left( L \right) = 26.5 \times \left[ {\frac{WT}{{90}}} \right]^{0.75}$ with a CV of 39%. Inter-compartmental clearance and peripheral volumes were 19.8 L/h and 22.3 L, respectively. Assuming co-administration of fosfomycin with meropenem, the dosing regimens of fosfomycin tested were 4 g, 6 g, and 8 g q8h as 3-h infusion.

Demographical characteristics of the critically ill patient population was simulated in 1:1 male:female ratio. Renal function of the population was a bimodal distribution as previously described^57^. The distributions for SCr to derive CLCr were based on two normal distributions for men and women. SCr distributions were 96 ± 14 μmol/L and 83 ± 12 μmol/L (mean ± SD) for male and female, respectively. These distributions were approximated from the serum creatinine distribution reported in the Hordaland Health Study^58^. The units were then converted to g/dL by dividing by 0.62. Age was uniformly distributed between the age of 41 to 74 years. CLCr was computed by the Cockcroft-Gault formula^59^. Body weights of patients were derived from height distributions of male and female adults as previously described^60^. The simulation for height was assumed to be normally distributed: male height was $176.3 \pm 0.17\sqrt {4482}$ cm (mean ± SD), and female height was $162.2 \pm 0.16\sqrt {4857}$ cm. The weight-height relationship was described by ${\text{WT}} = {\text{exp}}\left( {3.28 + 1.92{\text{ log HT}}} \right)$ and ${\text{WT}} = {\text{exp}}\left( {3.49 + 1.45{\text{ log HT}}} \right)$ for males and females, respectively. Simulated body weight was log-normally distributed such that $WT_{i} = WT \cdot \exp \eta$, wherein η is normally distributed with a mean of 0 and standard deviations (SD) of 0.14 and 0.17 for males and females, respectively, and *i* represents an individual.

The population PK models^61^ were simulated using RxODE package in R (v. 3.3.1), along with the demographical characteristics of the population.

### PD assessment using Monte Carlo simulation

The PD analyses carried out for 10,000 simulated concentration–time profiles were determined by a user-defined function in the R^62,63^. The percentage of each dosage interval in which free plasma concentration was within the MSW (%T_MSW_) and the percentage of each dosage interval in which free plasma concentration exceeds the MPC (%T_>MPC_) were estimated from the individual concentration–time profiles of polymyxin B, meropenem and fosfomycin in several dosing regimens. Summary statistics including mean and standard deviation were determined from the individual PD parameters.

The PD drug–drug interaction was taken into account in the checkerboard method^12,51^ through the reduction in MIC and MPC values in the following combinations: polymyxin B plus meropenem as a double combination; and polymyxin B plus meropenem and fosfomycin as a triple combination.

**Figure 4:**
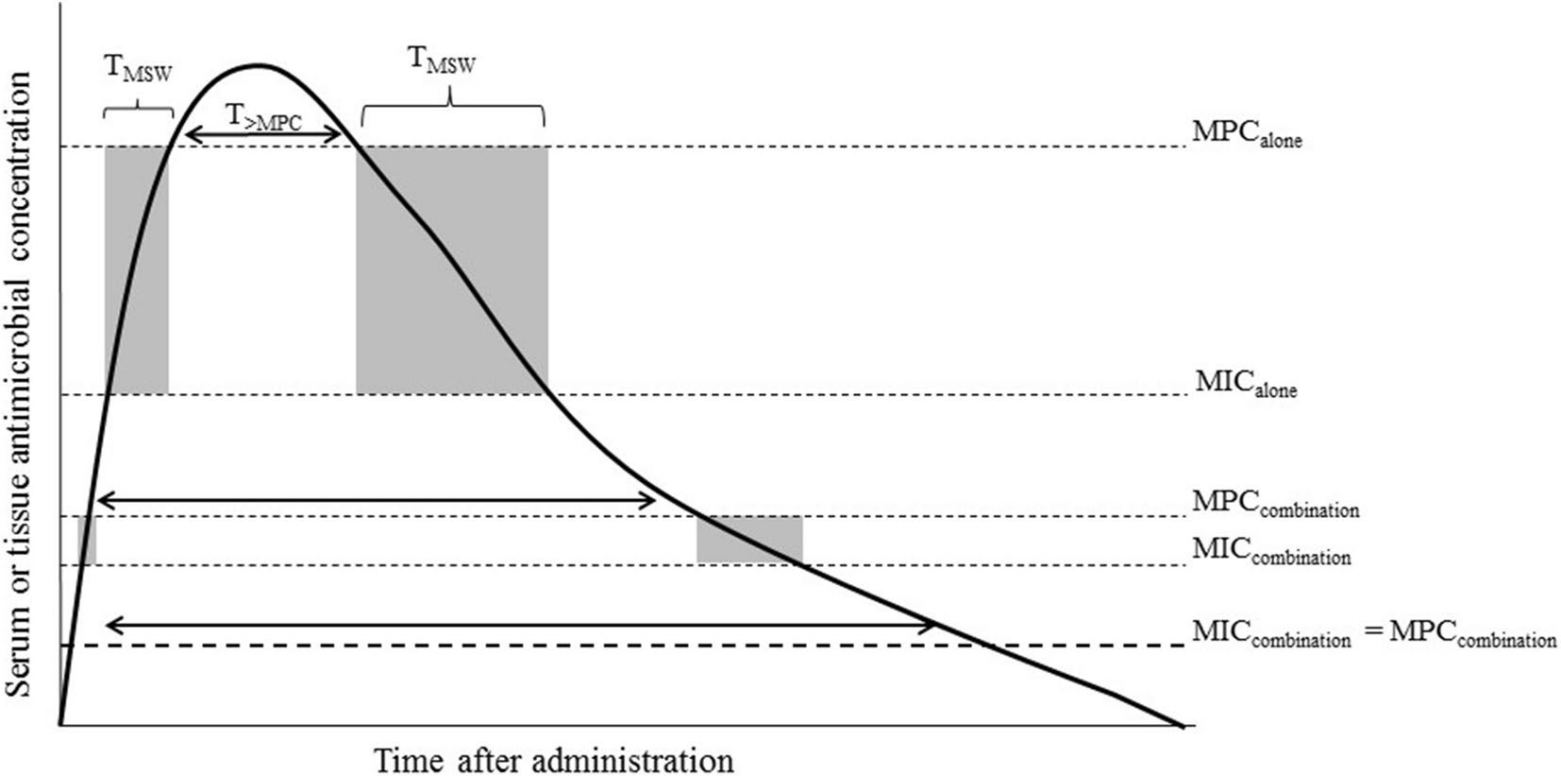
Schematic representation of the mutant selection window (MSW) of an antimicrobial agent alone and in combination with other drugs. As MIC and mutant prevention concentration (MPC) of the antimicrobial in the combination decrease, the time within the MSW (T_MSW_) decreases or is even eliminated when MIC and MPC values are equal. Essentially, the longer the time spent by an antibiotic within the MSW, the greater the opportunity for resistant mutants to be selectively amplified. Dashed lines indicate the MIC and MPC; double-headed arrow indicates the time that drug concentration exceeds the MPC (T_>MPC_); shaded region indicates the duration that drug concentration is within the MSW.

The PK/PD relationship for the three antimicrobial therapies as it relates to suppression of resistance has not yet been established. Both indices (%T_MSW_ and %T_>MPC_) of resistance prevention provided information regarding whether the polymyxin B dosing regimens in the combination (1) could reduce the time that drug concentration remains within the mutant selection window (%T_MSW_) and (2) could restrict the selection of resistant mutant when concentration above the MPC were achieved during treatment (%T_>MPC_). The schematic representation of the two PK/PD indices of resistance suppression in monotherapy and combination therapy is illustrated in Fig. 4.

### Ethical approval

This study was evaluated and approved by the Human Ethics Committee of State University of Maringa (COREA/COPEP no. 0447/2017).
